# Ossifying Fibroma of the Frontal Sinus: A Case Report and Literature Review

**DOI:** 10.7759/cureus.110025

**Published:** 2026-06-01

**Authors:** Alshaimaa Alqurashi, Mahmoud A Alreefi, Sumayya A Bafail, Omar S Almansouri, Ziyad S Alkhomri, Sami AlSowairi, Abdulmajeed Alahmari, Abdulrahman Alomari, Maha H Al-Attas, Elham S Bin Abbas

**Affiliations:** 1 Otolaryngology - Head and Neck Surgery, King Fahad Armed Forces Hospital, Jeddah, SAU; 2 Otolaryngology - Head and Neck Surgery, Faculty of Medicine, King Abdulaziz University, Rabigh, SAU; 3 Otolaryngology - Head and Neck Surgery, East Jeddah Hospital, Jeddah, SAU; 4 Otolaryngology - Head and Neck Surgery, King Fahad General Hospital, Jeddah, SAU; 5 Otolaryngology - Head and Neck Surgery, College of Medicine, King Saud Bin Abdulaziz University for Health Sciences, Jeddah, SAU; 6 Oral and Maxillofacial Surgery, King Fahad Armed Forces Hospital, Jeddah, SAU; 7 Otolaryngology - Head and Neck Surgery, Saudi German Hospital, Jeddah, SAU; 8 Pathology and Medical Laboratory, King Fahad Armed Forces Hospital, Jeddah, SAU

**Keywords:** endoscopic sinus surgeries, external approach, frontal sinus surgery, headache, orbital pain, ossifying fibroma, surgical case reports

## Abstract

Ossifying fibroma is a rare benign fibro-osseous tumor that rarely arises from the paranasal sinuses and tends to involve the frontal sinus more frequently than any other. Here, we report a case of a 32-year-old woman presenting with a 1.5-year history of facial pain on the right side of her eye along with proptosis. Radiographic findings revealed total opacification and bony changes in the right frontal sinus. The patient was treated with endoscopic sinus surgery for the purpose of performing a biopsy, followed by resection using the external approach. Ossifying fibroma was confirmed histologically. It is important to consider the possibility of ossifying fibroma when faced with a mass in the frontal sinus and that a radical excision is required for better results.

## Introduction

Ossifying fibroma is a bone tumor composed of cementum-like tissue; it is a benign tumor and is classified as a fibro-osseous lesion [1,2]. It usually affects females in their second to fourth decades of life, with the possibility of affecting any age group [1]. The lesion typically arises in the molar region of the mandible, whereas its occurrence in the paranasal sinuses, particularly the frontal sinus, is exceptionally rare [3-5]. Sun et al. reported only one case of frontal sinus ossifying fibroma over a five-year period in the Department of Otolaryngology at Gongli Hospital (Shanghai, China), emphasizing the rarity of this condition [5]. Due to its indolent growing pattern and wide variety of presentations, ossifying fibroma can be overlooked [6]. The clinical presentation may vary depending on the lesion site, but generally, patients may present with a painless bony mass [6,7]. In a series by Chang et al., 26 out of 28 cases involved the mandible, and the remaining two were maxillary lesions. The most common clinical presentation was bony swelling [7]. Radiographically, this benign tumor starts small and radiolucent, and as it gets bigger and more mature, it tends to be more radiopaque [8]. Histologically, ossifying fibroma consists of a mixture of lamellar and woven bone with cementum-like material in a fibrous stroma [7]. With the risk of recurrence and the rare malignant transformation, radical surgical excision is required to remove this tumor [9,10]. This case report highlights an ossifying fibroma localized to the frontal sinus.

## Case presentation

A 32-year-old female, previously healthy, not known to have any allergy, presented to the family medicine clinic with a chief complaint of intermittent right eye and supraorbital pain for 1.5 years that progressed to proptosis over the last four months. She also reported intermittent left-sided headache, left-sided nasal obstruction, sneezing, and clear rhinorrhea. Additionally, she mentioned a history of episodic subjective fever and fatigue for one year. There was no history of weight loss, night sweats, epistaxis, or loss of smell. She was referred to the neurology department for further evaluation.

Magnetic resonance imaging (MRI) showed complete opacification of the right frontal sinus extending to the anterior ethmoid air cells, with high T1, low T2, and intermediate fluid-attenuated inversion recovery (FLAIR) signal intensity. There was mucosal thickening of the left frontal sinus with some central high T1 signal-intense component. Bone remodelling was noted with possible extension to the superior aspect of the right orbit within the extracoronal space (Figures 1-2). Based on these findings, she was referred to the otorhinolaryngology department with a presumptive diagnosis of frontal sinus fungal infection.

**Figure 1:**
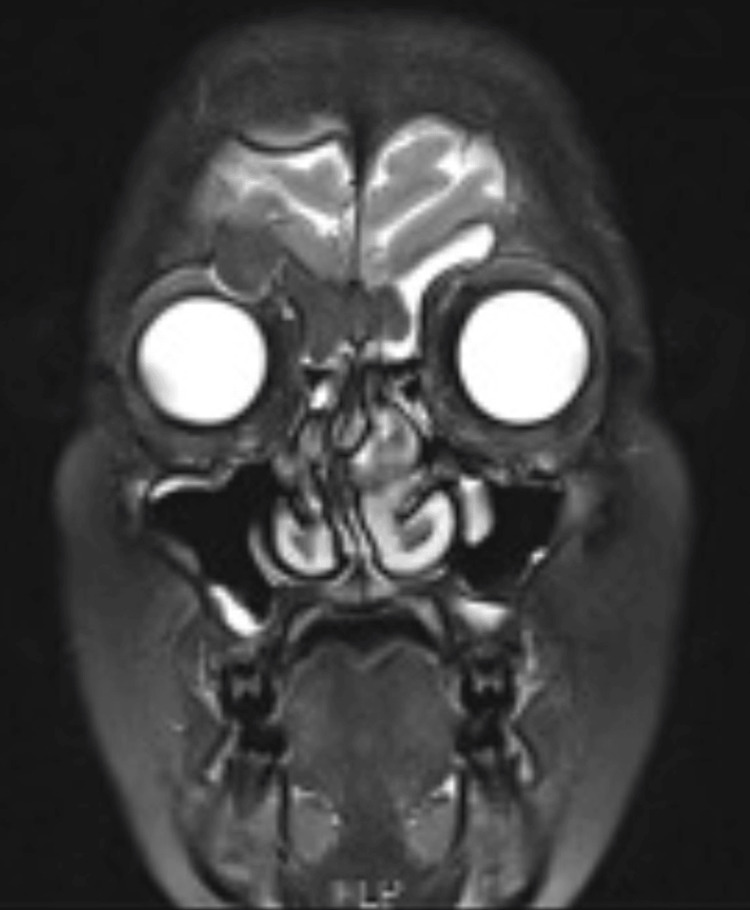
Coronal T1-weighted MRI showing complete opacification of the right frontal sinus with high signal intensity, extending into the anterior ethmoid air cells and causing orbital displacement.

**Figure 2:**
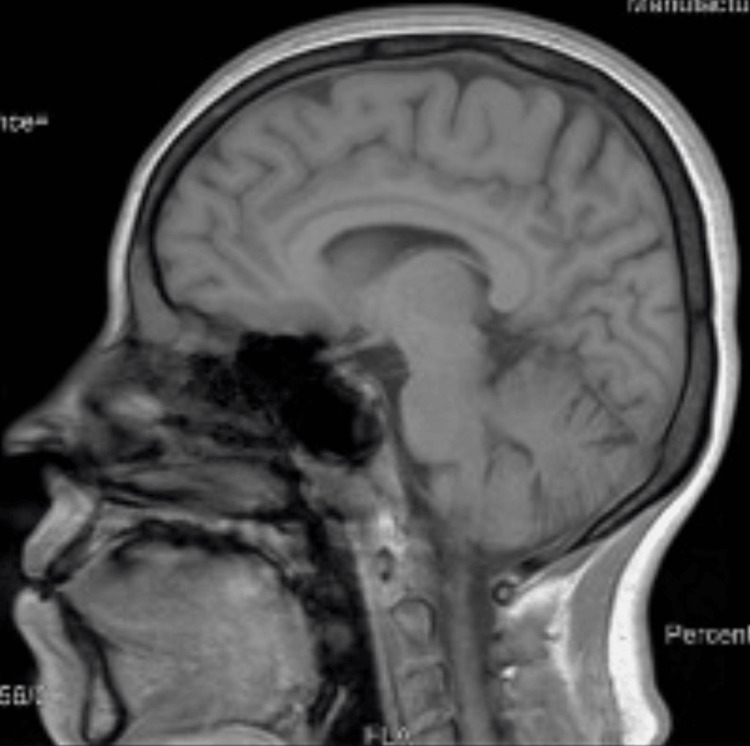
Sagittal MRI demonstrating the extent of the frontal sinus lesion with characteristic signal intensity and its relationship to the superior orbital wall.

Upon examination, the patient had right-sided exophthalmos. Nasal endoscopy revealed no polyps or discharge. Computed tomography (CT) of the paranasal sinuses confirmed complete opacification of the right frontal sinus with hyperdense material, extending to the right anterior ethmoid air cells, and showed homogeneous enhancement on post-contrast image. Bony remodelling was noted at the cribriform plate, right lamina papyracea, and the right superior orbital wall. There was mucosal thickening of the left frontal sinus with a central area of hyperdense material. Additionally, there was mucosal thickening involving both maxillary and sphenoidal sinuses (Figures 3-5).

**Figure 3:**
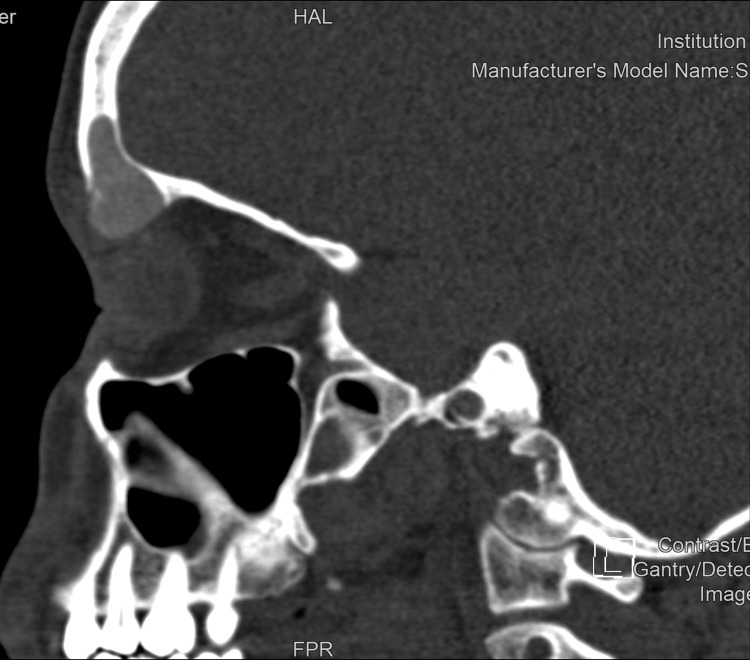
Sagittal non-contrast CT scan of the paranasal sinuses showing a hyperdense mass completely filling the right frontal sinus with associated bony remodeling of the sinus walls.

**Figure 4:**
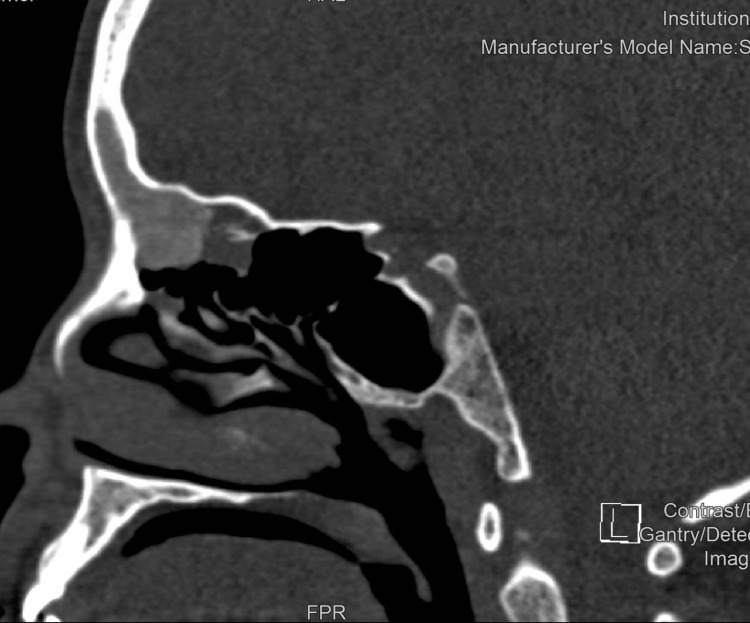
Sagittal post-contrast CT image illustrating the homogeneous enhancement of the right frontal sinus lesion and its proximity to the anterior cranial base.

**Figure 5:**
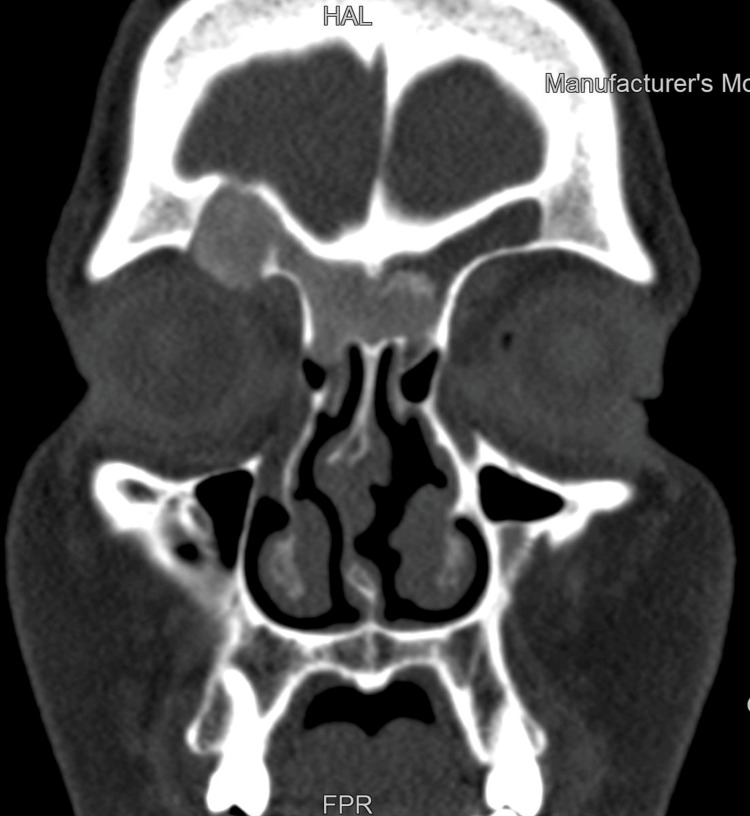
Coronal CT scan showing the expansile nature of the ossifying fibroma within the right frontal sinus, causing thinning and remodeling of the lamina papyracea and superior orbital rim.

The patient underwent bilateral endoscopic sinus surgery (ESS). On the left side, a partial middle turbinate resection and turbinectomy were carried out, followed by a maxillary antrostomy, frontal sinusotomy, and complete ethmoidectomy. On the right side, uncinectomy and maxillary antrostomy were done. A spongy mass completely filling the right frontal sinus was identified and biopsied. Histopathology revealed variable mineralization from woven to lamellar bone along with osteoid formation. The lesion's well-circumscribed nature and clinical presentation were consistent with an ossifying fibroma (Figure 6).

**Figure 6:**
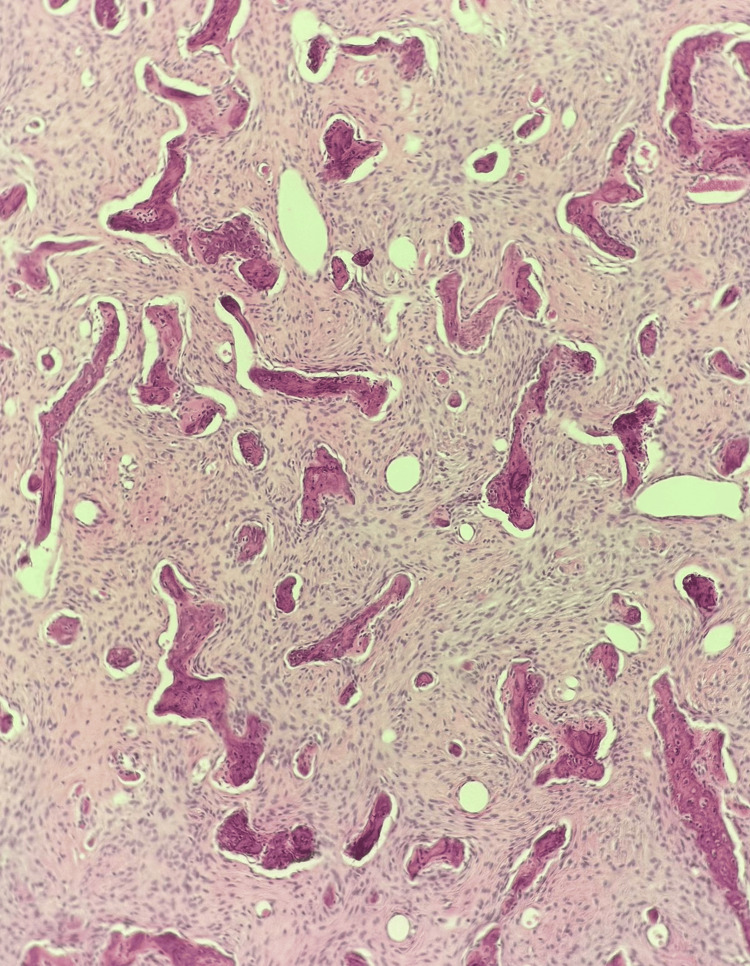
Histopathological section (H&E stain) showing irregular bony trabeculae with varying degrees of mineralization and osteoid formation embedded within a cellular fibrous stroma, characteristic of ossifying fibroma.

One month later, definitive removal of the lesion was performed via an external approach. Local anesthesia with epinephrine was administered, and a skin incision was made using a 15-blade scalpel. Blunt dissection was done using finger techniques until the suborbital rim and bone were reached. Suborbital bundle was identified bilaterally, periosteal incision was done, the bone was exposed, a window was created to open the sinus, a lesion was identified bilaterally and removed, and open reduction and internal fixation (ORIF) of the bone was conducted using two plates, one with five holes and another with 16 holes, and secured with nine screws (15 mm and 9 mm). Hemostasis was achieved, and closure was completed in layers using 3-0 Vicryl sutures. A surgical drain was inserted, skin staplers were applied, and a pressure dressing was placed.

## Discussion

The first case of ossifying fibroma was described in 1872. At that time, both ossifying fibroma and fibrous dysplasia were considered one entity. Subsequent advances in clinical, radiologic, and histopathologic characterization have distinguished the two entities [11]. Ossifying fibroma is a bone-derived tumor that is indolent in growth, whereas fibrous dysplasia is a hyperplastic bone lesion caused by bone mesenchymal dysplasia [12]. Ossifying fibroma usually affects females between 20 and 40 years of age. It mostly arises in the mandible and maxilla; however, it has been reported in the literature to occur in other sites like the craniofacial bones [13,14].

The cause of the disease is unknown. Current theories suggest the lesion is derived from bone tissue during adolescence, with trauma serving as a precipitating factor [12,13]. Most lesions are asymptomatic and found incidentally. Once the lesion gets bigger, it starts to cause symptoms specific to its site. The most frequently reported symptom is painless bony swelling [3,6].

In our case, the patient had an ossifying fibroma in the frontal sinus, a rare site, presenting with headache, eye pain, and proptosis. Her age (32 years) was more consistent with a conventional ossifying fibroma than with the aggressive juvenile variant, which typically occurs before age 15 [15]. Diagnostic evaluation included CT of the paranasal sinuses to assess osseous changes and MRI to investigate potential soft tissue extension [5,6]. Radiographically, ossifying fibroma typically appears as a round or oval, high-density lesion with a uniform internal pattern and well-defined borders. In contrast, fibrous dysplasia demonstrates an even-density mass with poorly defined margins, often described as having a "frosted glass" or "ground glass" appearance [5].

Histopathologically, an ossifying fibroma appears as a fibro-osseous lesion with clear boundaries in a fibrous stroma. On the other hand, fibrous dysplasia is characterized by irregular bone trabeculae, disorganized intersecting collagen fibers, numerous osteoclasts within the bone matrix, and a lack of clear boundaries between the lesion and surrounding bone [5].

Although histologically slow growing, ossifying fibromas can cause significant morbidity when expanding into adjacent structures. Involvement of the orbit can result in proptosis, as occurred in our patient [10]. With a malignancy transformation rate of 0.4%-0.5% and a possibility of recurrence, complete surgical resection is required [9,10]. There are many surgical approaches that can be utilized depending on both the lesion site and extent [16]. In our case, initial ESS was performed for a presumptive diagnosis of allergic fungal sinusitis, during which the lesion was unexpectedly encountered. Definitive removal was achieved via a bilateral lower eyelid (BLE) external approach, providing adequate exposure and facilitating complete excision. Recurrence following incomplete excision has been documented to occur between six months and seven years postoperatively [9]. Alternative treatment modalities, such as radiotherapy, are not advised due to the potential risk of inducing malignant transformation, specifically osteosarcoma [17]. In a case analogous to ours, Sun et al. documented a 46-year-old female patient who presented with forehead swelling and dizziness. CT scan similarly revealed a lesion in the right frontal sinus, suggestive of a fungal infection. During surgical intervention, the tumor was found to be encapsulated, resembling bone tissue with well-defined boundaries. Following complete surgical resection, the patient's symptoms resolved. The patient was monitored for five years postoperatively, during which no recurrence was observed [5].

This case contributes to the limited body of literature on ossifying fibromas of the frontal sinus (Table 1) and highlights the necessity of considering this condition in the differential diagnosis of frontal sinus lesions, particularly when imaging reveals bony remodeling or hyperdensity. Awareness of such rare presentations facilitates prompt diagnosis and definitive surgical intervention, thereby ensuring an excellent prognosis [5].

**Table 1:** Comparative analysis of ossifying fibroma cases in paranasal sinuses.

Study	Age/Sex	Location	Symptoms	Treatment	Outcome
Sun et al. (2013) [5]	46/F	Right frontal sinus	Forehead swelling, dizziness, eyeball dislocation	Frontal sinus fenestration, tumor resection, nasofrontal duct reconstruction	No recurrence at 5 years
Babaheidarian et al. (2024) [18]	50/M	Left frontal sinus	Left eye proptosis, headaches	Open surgical resection	Good prognosis
Chevreux et al. (2023) [19] - Case 1	14/F	Right ethmoid sinus, orbital extension	Nasal obstruction, exophthalmos	Endoscopic resection	Recurrence at 1 year, revision surgery, stable remnants
Chevreux et al. (2023) [19] - Case 2	56/M	Nasal septum, skull base	Nasal obstruction, dacryocystitis	Chemotherapy, endoscopic resection, radiotherapy	No recurrence at 3 years
Chevreux et al. (2023) [19] - Case 3	30/F	Skull base, sphenoid sinus	Asymptomatic (incidental)	Endoscopic biopsy/partial excision	Stable residual tumor
Almusallam et al. (2025) [6]	32/F	Frontal sinus	Eye pain, proptosis, headache	ESS, followed by an external approach	Excellent prognosis
Budu et al. (2011) [20]	60/F	Frontal sinus	Diffuse, intermittent headache	Surgical resection	Recurrence common in sinuses
Present Case	32/F	Right frontal sinus	Right eye/supraorbital pain, proptosis, headache	ESS (biopsy), followed by external approach	Excellent prognosis

## Conclusions

Overall, the ossifying fibroma of the frontal sinus represents an uncommon benign fibro-osseous neoplasm that can present with vague symptoms, hence posing diagnostic dilemmas. In light of the above case, there is a need for a high degree of clinical suspicion during the assessment of masses within the frontal sinus, particularly where the radiographic findings depict bony remodeling and hyperdense masses. Although this condition progresses slowly, the morbidity associated with the condition may be high due to the expanding nature and possible involvement of surrounding tissues, which is evident through the orbital displacement and proptosis shown in this case. Surgical excision forms the core of the management strategy as it will minimize any chances of recurrence. Early diagnosis and proper surgical treatment are key to ensuring a good outcome. This study contributes to the few studies on ossifying fibroma of the frontal sinus and highlights the importance of identifying its uncommon presentations.
